# Correction: The shadow of the family: Historical roots of social trust in Europe

**DOI:** 10.1371/journal.pone.0318590

**Published:** 2025-01-28

**Authors:** Maria Kravtsova, Aleksey Oshchepkov, Christian Welzel

There are errors in the Author Contributions. The correct contributions are:

**Conceptualization:** Maria Kravtsova, Aleksey Oshchepkov, Christian Welzel.

**Data curation:** Maria Kravtsova.

**Formal analysis:** Maria Kravtsova, Aleksey Oshchepkov.

**Methodology:** Maria Kravtsova, Aleksey Oshchepkov.

**Supervision:** Christian Welzel.

**Visualization:** Maria Kravtsova.

**Writing–original draft:** Maria Kravtsova, Aleksey Oshchepkov.

**Writing–review & editing:** Christian Welzel.
