# Supplementary material for: The shadow of the family: Historical roots of social trust in Europe
Source: PLoS One. 2024 Feb 12;19(2):e0295783. doi: 10.1371/journal.pone.0295783 (PMC10861049; doi:10.1371/journal.pone.0295783)
Supplement: S1 File — (DOCX) [file pone.0295783.s001.docx]

**S1 File. Data sources and additional sample description.**

**Table S1.1 Sources of historical data on family structure.**

| **Historical state** | **Contemporary state** | **Source** |
| --- | --- | --- |
| Albania | Albania | Albanian census 1918, Mosaic Project https://censusmosaic.demog.berkeley.edu |
| Bulgaria | Bulgaria | Mosaic project, https://censusmosaic.demog.berkeley.edu |
| France | France | 1. INSEE, Recencements de 1851 à 1921 (données de la SGF) http://www.insee.fr/fr/service/bibliotheque/tableaux_sgf/tableaux.asp?domaine=rec  2. Mosaic project, https://censusmosaic.demog.berkeley.edu |
| Germany | Germany, Poland | 1. Vierteljahrshefte zur Statistik des Deutschen Reichs für das Jahr 1873”, Verlag des Königlich Preussischen Statistischen Bureaus, 1874, provided by the DFG project 'Digitisation of the Statistics of the German Reich [A.F.] 1873-1883]'  2. Mosaic project, https://censusmosaic.demog.berkeley.edu |
| England and Wales | England and Wales | IPUMS, International |
| Habsburg Empire (Austrian part) | Austria, Szech Republik, Slovenia, Poland, Croatia, Northern Italy. | 1. Household statistic, 1910 Österreichische Statistik, N. F., vol. 4, no. 3, p. 1. Wien 1918.  2. Mosaic project, https://censusmosaic.demog.berkeley.edu |
| Habsburg Empire (Hungarian part) | Hungary, Slovakia, Romania, Croatia. | 1. évi népszámlálás. [Census 1910]. Vol. 6. Végeredmények összefoglalása. [Summary of results]. 1920 2. Mosaic project, https://censusmosaic.demog.berkeley.edu |
| Italy | Italy | “Sommario di Statistiche Storiche dell’Italia (1861-1975)”, Instituto Centrale di Statistica, Roma, 1976. |
| Russian Empire | Russia, Poland, CIS states | 1. Troinitskiy N. (1899-1904) The first universal census of Russian Empire, 1897. Central Statistical Agency of the Ministry of Internal Affairs (Первая всеобщая перепись населения Российской Империи, 1897 г Центральный статистический комитет МВД).  2. Mosaic project, https://censusmosaic.demog.berkeley.edu |
| Scotland | Scotland | IPUMS, International |
| Serbia | Serbia | Statistique du Royaume de Serbie, Belgrade, Imprimerie de l'etat du Royaume de Serbie, vol. XXIII-XXIV, 1903-1905 |
| Sweden | Sweden | IPUMS, International |

**Table S1.2. Availability of historical explanatory variables**

| State | Province | Year | mean_HH no one person | one person HH | Mean HH (mean HH no children) | Mean kin group no one person hh | Children/adults | HH adult children | HH married son | Elderly not HH head | Lateral relatives | Vertical family extensions (all variables) | | Servants | women20_29 | female hh heads (G) | young brides (G) | wives older (G) | female non kin (G) | Population  density |
| --- | --- | --- | --- | --- | --- | --- | --- | --- | --- | --- | --- | --- | --- | --- | --- | --- | --- | --- | --- | --- |
| Albania |  | 1923 |  |  |  |  |  |  |  |  |  |  |  | |  |  |  |  |  | X |
| Albania |  | 1918 | X | X | X | X | X | X | X | X | X | X | X | | X | X | X | X | X |  |
| Bulgaria |  | 1877-1947 | |  |  |  |  |  |  |  |  |  |  | |  | X | X | X | X |  |
| Bulgaria |  | 1881 |  |  |  |  |  |  |  |  |  |  |  | |  |  |  |  |  | X |
| England and Wale |  | 1881 | X | X | X | X | X | X | X | X | X | X | X | | X | X | X | X | X | X |
| France |  | 1891 |  |  |  |  |  |  |  |  |  |  |  | |  |  |  |  |  |  |
| France |  | 1886 | X | X | X | X | X | X | X | X | X | X | X | | X |  |  |  |  |  |
| France |  | 1881 |  |  |  |  |  |  |  |  |  |  |  | |  |  |  |  |  | X |
| France |  | 1872 |  |  |  |  |  |  |  |  |  |  |  | |  |  |  |  |  |  |
| France |  | 1846 |  |  |  |  |  |  |  |  |  |  |  | |  | X | X | X | X |  |
| German Empire |  | 1871 | X | X |  |  |  |  |  |  |  |  |  | | X |  |  |  |  | X |
| German Empire | Prussia | 1871 |  |  |  |  |  |  |  |  |  |  |  | |  |  |  |  |  |  |
|  | Ostpreussen, Danzig | 1695-1772 | |  |  |  |  |  |  |  |  |  |  | |  | X | X | X | X |  |
|  | Posen | 1666-1809 | |  |  |  |  |  |  |  |  |  |  | |  | X | X | X | X |  |
|  | Bromberg | 1766-1792 | |  |  |  |  |  |  |  |  |  |  | |  | X | X | X | X |  |
|  | Breslau, Liegnitz, Oppeln | 1747-1805 | |  |  |  |  |  |  |  |  |  |  | |  | X | X | X | X |  |
| Habsburg Empire (Austrian part) |  | 1900 | X | X |  |  |  |  |  |  |  |  |  | | X |  |  |  |  | X |
| Austrian provinces | Silesia | 1747-1805 | |  |  |  |  |  |  |  |  |  |  | |  | X | X | X | X |  |
|  | Styria | 1910 |  |  |  |  |  |  |  |  |  |  |  | |  | X | X | X | X |  |
|  | Galizia | 1747-1805 | |  |  |  |  |  |  |  |  |  |  | |  | X | X | X | X |  |
| Hungary |  | 1910 |  |  |  |  |  |  |  |  |  |  |  | |  |  |  |  |  | X |
| Habsburg Empire (Hungarian part) |  | 1869 | X | X | X | X | X | X | X | X | X | X | X | | X | X | X | X | X |  |
|  | Kingdom of Croatia | 1910 |  |  |  |  |  |  |  |  |  |  |  | |  |  |  |  |  | X |
| Italy |  | 1901 | X | X |  |  |  |  |  |  |  |  |  | | X | X | X | X | X | X |
| Italy |  | 1900 |  |  |  |  |  |  |  |  |  |  |  | |  |  |  |  |  |  |
| Polish provinces | Warshau | 1666-1809 | |  |  |  |  |  |  |  |  |  |  | |  | X | X | X | X |  |
|  | Warshau, Plotsk | 1766-1792 | |  |  |  |  |  |  |  |  |  |  | |  | X | X | X | X |  |
|  | Kalissk | 1790-1792 | |  |  |  |  |  |  |  |  |  |  | |  | X | X | X | X |  |
|  | Kelitsk, Petrokovsk | 1789-1792 | |  |  |  |  |  |  |  |  |  |  | |  | X | X | X | X |  |
|  | Lyublinsk, Sedletsk | 1791-1792 | |  |  |  |  |  |  |  |  |  |  | |  | X | X | X | X |  |
| Romania |  | 1859 |  |  |  |  |  |  |  |  |  |  |  | |  |  |  |  |  | X |
| Romania |  | 1838 | X | X | X | X | X | X | X | X | X | X | X | | X | X | X | X | X |  |
| Russian Empire |  | 1897 | X | X |  |  |  |  |  |  |  |  | X | | X |  |  |  |  | X |
| Russian Empire | Vilna, Minsk | 1768-1804 | |  |  |  |  |  |  |  |  |  |  | |  | X | X | X | X |  |
|  | Volhinya | 1791-1792 | |  |  |  |  |  |  |  |  |  |  | |  | X | X | X | X |  |
|  | Volhinya, Kiev | 1791 |  |  |  |  |  |  |  |  |  |  |  | |  | X | X | X | X |  |
|  | Minsk | 1795 |  |  | X | X | X | X | X | X | X | X | X | |  | X | X | X | X |  |
| Scotland |  | 1881 | X | X |  |  |  |  |  |  |  |  |  | | X | X | X | X | X | X |
| Serbia |  | 1900 | X | X |  |  |  |  |  |  |  |  |  | | X | X | X | X | X | X |
| Sweden |  | 1900 |  |  |  |  |  |  |  |  |  |  |  | |  | X | X | X | X |  |
| Sweden |  | 1880 | X | X | X | X | X | X | X | X | X | X | X | | X |  |  |  |  | X |

Note: Time periods (ex.1666-1809) mean that different localities within one historical region were studied at different time points. Geographic indicators and Medieval Church exposure

based on GIS analysis are time invariant variables and are available for all the regions.

**Table S1.3: Regional geographic and historical controls**

| **Variable** | **Source** |
| --- | --- |
| Population density | Rothenbacher, F. (2017). *The European Population, 1850-1945*. Springer.Rothenbacher, F. (2016). The Central and East European Population since 1850. Springer. |
| Cool Water Index (CWI) | To calculate CWI we used methodology elaborated by Welzel, C., Alexander, A. C., & Klasen, S. 2018. The Cool Water Effect. [www.researchgate.net](http://www.researchgate.net). Data on temperature and precipitations is based on information from meteorological stations www.meteoblue.com |
| Caloric suitability of land for agriculture | Galor & Özak, 2016 |
| Ruggedness of the terrain | Nunn & Puga, 2012 |
| Proximity to the waterways (distance from the centroid of the region to the nearest point on the coast or on the main river) | [Global Self-consistent, Hierarchical, High-resolution Geography Database (GSHHG)](http://www.soest.hawaii.edu/pwessel/gshhg/)**,** https://www.ngdc.noaa.gov/mgg/shorelines/ |
| Prevalence of land suitability for hunting and gathering over agriculture | Beck & Sieber, 2010 |
| Exposure to the medieval Catholic church | Schulz et al., 2019b |
