# Supplementary material for: The shadow of the family: Historical roots of social trust in Europe
Source: PLoS One. 2024 Feb 12;19(2):e0295783. doi: 10.1371/journal.pone.0295783 (PMC10861049; doi:10.1371/journal.pone.0295783)
Supplement: S2 File — (DOCX) [file pone.0295783.s002.docx]

**S2 File. Principal Components Analysis results**

**Table S2.1:** The results of Principal Components Analysis (PCA) for constructing generational and gender hierarchy indices.

|  | Generational hierarchy (PC1) | Gender hierarchy (PC1) |
| --- | --- | --- |
| Prevalence of vertical hh headed by son | -0.800 |  |
| Vertical hh, all | 0.800 |  |
| Female hh heads (G) |  | 0.757 |
| Single women 20-29 |  | -0.941 |
| Young brides (15-19) (G) |  | 0.936 |
| Wives older (G) |  | 0.902 |
| Females non kin (G) |  | 0.822 |

Note: On this table you can see the results of two separate PCA that were used for constructing generational and gender hierarchy indices. Entries are

correlation coefficients for variables loaded on the first principal component in each PCA. Gender hierarchy items labeled with (G) come from

Gruber and Szoltysek’s (2016) Patriarchy index. They were originally rescaled in a way that higher scores mean more patriarchy.
