# Supplementary material for: The shadow of the family: Historical roots of social trust in Europe
Source: PLoS One. 2024 Feb 12;19(2):e0295783. doi: 10.1371/journal.pone.0295783 (PMC10861049; doi:10.1371/journal.pone.0295783)
Supplement: S3 File — (DOCX) [file pone.0295783.s003.docx]

**S3 File. OLS regressions results**

**Table S3.1**: OLS regression of the contemporary out-group trust on historical family indicators (Gender hierarchy index excludes the proportion of

female household heads).

| DV: out-group trust (LiTS, 2010) | (1) | (2) | | (3) | (4) | (5) | (6) | (7) | (8) | (9) |
| --- | --- | --- | --- | --- | --- | --- | --- | --- | --- | --- |
| Generational hierarchy | -0.684*** |  | | -0.686*** | -0.731*** | -0.741*** | -0.735*** | -0.784*** | -0.715*** | -0.727*** |
|  | (0.179) |  | | (0.183) | (0.188) | (0.204) | (0.208) | (0.244) | (0.190) | (0.202) |
| Gender hierarchy (excl. female hh heads) |  | -0.0240 | | 0.0403 | -0.0161 | -0.112 | 0.149 | 0.365 | -0.121 | 0.210 |
|  |  | (0.261) | | (0.247) | (0.298) | (0.289) | (0.347) | (0.351) | (0.309) | (0.399) |
| Mean HH |  |  | |  |  | -0.246 |  |  |  |  |
|  |  |  | |  |  | (0.191) |  |  |  |  |
| Mean HH no children |  |  | |  |  |  |  |  | -0.200 |  |
|  |  |  | |  |  |  |  |  | (0.236) |  |
| Mean HH no one person HH |  |  | |  |  |  | 0.0908 |  |  |  |
|  |  |  | |  |  |  | (0.217) |  |  |  |
| Mean kin group size no one person HH |  |  | |  |  |  |  |  |  | 0.155 |
|  |  |  | |  |  |  |  |  |  | (0.229) |
| One person HH |  |  | |  |  |  | 3.572 |  |  | 3.778 |
|  |  |  | |  |  |  | (2.436) |  |  | (2.441) |
| Children/adults |  |  | |  |  |  | -1.325 | -1.845 |  | -1.569 |
|  |  |  | |  |  |  | (1.565) | (1.511) |  | (1.565) |
| Lateral no vertical |  |  | |  |  |  |  | 2.443 |  |  |
|  |  |  | |  |  |  |  | (3.141) |  |  |
| Servants |  |  | |  |  |  |  |  |  | 0.653 |
|  |  |  | |  |  |  |  |  |  | (1.893) |
| Population density |  |  | |  | -2.20e-05 | -7.72e-05 | -0.000159 | -2.41e-05 | -5.39e-05 | -0.000158 |
|  |  |  | |  | (3.75e-05) | (5.60e-05) | (0.000103) | (4.29e-05) | (5.41e-05) | (0.000104) |
| CWI |  |  | |  | -1.385 | -1.273 | -1.244 | -1.153 | -1.352 | -1.274 |
|  |  | |  |  | (1.146) | (1.050) | (1.046) | (1.038) | (1.102) | (1.063) |
| Individual level controls | YES | | | | | | | | | |
| Historical country FE | YES | | | | | | | | | |
| Observations | 4,042 | 4,042 | | 4,042 | 4,042 | 4,042 | 4,042 | 3,801 | 4,042 | 4,042 |
| R-squared | 0.233 | 0.225 | | 0.233 | 0.234 | 0.235 | 0.237 | 0.243 | 0.235 | 0.237 |

Note: Standard errors are clustered at the level of historical sub-national regions (94 regions). Entries are unstandardized regression coefficients with standard errors in parentheses. *p < .05; **p < .01; ***p < .001

**Table S3.2:** OLS regression of the contemporary level of out-group trust on mean household size and the proportion of never married women in the age

group 20-29 (extended sample and core sample).

|  | Extended sample | | Core sample | |
| --- | --- | --- | --- | --- |
| DV: out-group trust (LiTS, 2010) | (1) | (2) | (3) | (4) |
| % of single women 20-29 | 1.620** | 0.824 | -0.713 | -0.776 |
|  | (0.673) | (0.705) | (1.214) | (1.291) |
| Population density |  | 1.54e-05 |  | 7.19e-06 |
|  |  | (1.81e-05) |  | (2.44e-05) |
| CWI |  | 1.736 |  | -0.433 |
|  |  | (1.509) |  | (1.192) |
| Individual level controls | YES | | | |
| Historical country FE | YES | | | |
| Observations | 22,860 | 22,860 | 4,042 | 4,042 |
| R-squared | 0.098 | 0.107 | 0.225 | 0.225 |

Note: For comparison we estimate the same model specification on a core sample in order to observe how the coefficients change with the increase of the sample size.

Standard errors are clustered at the level of historical sub-national regions (292 regions). *** p<0.01, ** p<0.05, * p<0.1
