# Supplementary material for: The shadow of the family: Historical roots of social trust in Europe
Source: PLoS One. 2024 Feb 12;19(2):e0295783. doi: 10.1371/journal.pone.0295783 (PMC10861049; doi:10.1371/journal.pone.0295783)
Supplement: S1 Fig — (DOCX) [file pone.0295783.s004.docx]

**S4 Figure. Validation of LiTS regional measure of out-group trust**

**Fig. S4:** Correlation between out-group trust in the Russian regions coming from

LiTS survey (2010) and from the survey “Values in the Russian Regions”, (2019).


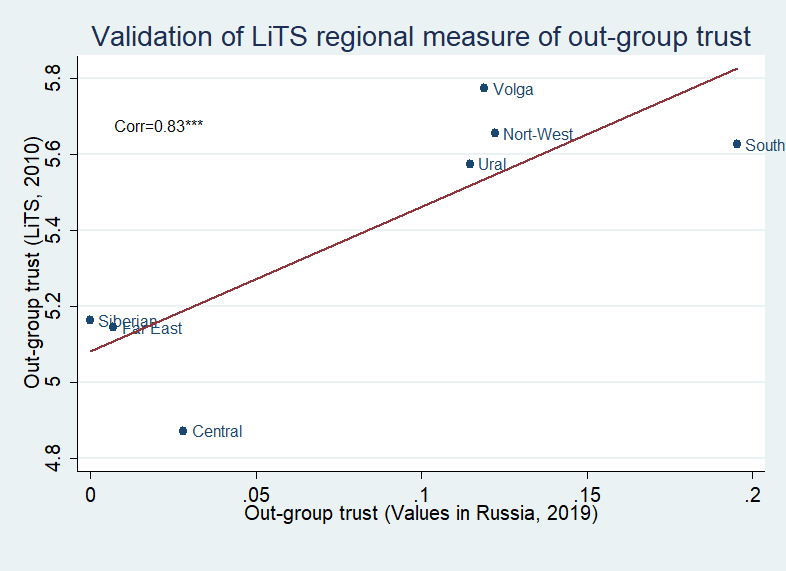


Note: To construct out-group trust index we use in both surveys two identical items 1) trust people you meet for the first time;

2) trust people of another nationality. We do not use the third item (trust people of another religion) because it was not available in the Russian survey.

We rescale out-group trust from the Russian survey so that higher values mean more trust (-1* (observed score-max. score)) in order to adjust it to the LiTS scale.
