# Supplementary material for: The shadow of the family: Historical roots of social trust in Europe
Source: PLoS One. 2024 Feb 12;19(2):e0295783. doi: 10.1371/journal.pone.0295783 (PMC10861049; doi:10.1371/journal.pone.0295783)
Supplement: S2 Fig — (DOCX) [file pone.0295783.s005.docx]

**Figure S5. Vertical and lateral extensions across 94 regions.**

**Fig. S5:** Incidence of vertical and lateral extensions across 94 regions (core sample) of historical Western and Eastern Europe.


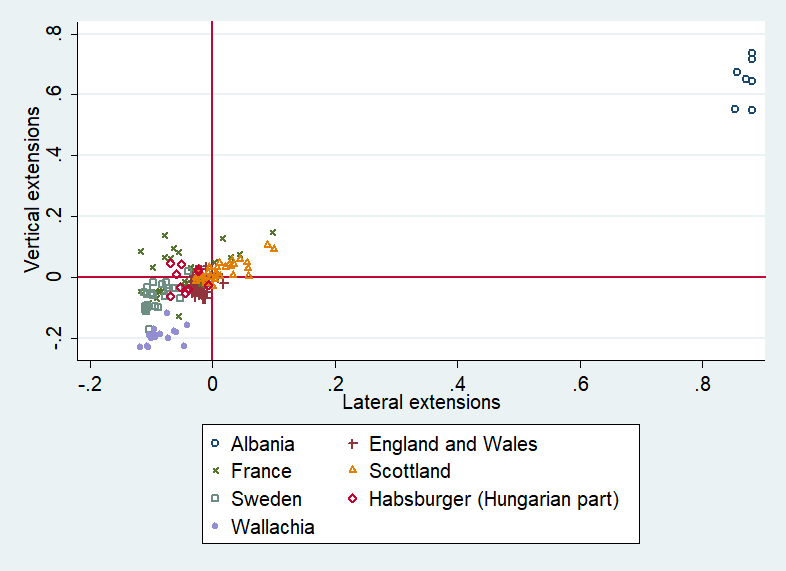


Note: Both variables were rescaled so that 0 is the mean value. On the x-axis is shown the percentage of historical households that have lateral (horizontal) extensions. On the y-axis is shown the percentage of historical vertically extended households headed by the oldest man in the household.
