# Supplementary material for: The shadow of the family: Historical roots of social trust in Europe
Source: PLoS One. 2024 Feb 12;19(2):e0295783. doi: 10.1371/journal.pone.0295783 (PMC10861049; doi:10.1371/journal.pone.0295783)
Supplement: S5 Fig — (DOCX) [file pone.0295783.s008.docx]

**Figure S8.** **Generational hierarchy and lateral extensions excluding Albania**

**Fig. S8:** Incidence of generational hierarchy and lateral extensions across historical Western and Eastern Europe excluding Albania.


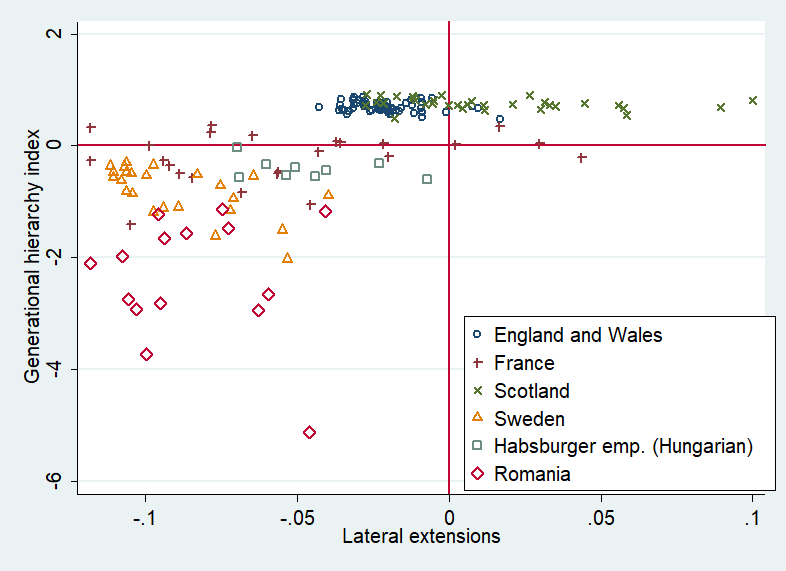


Note: Both variables were rescaled so that 0 is the mean value. On the x-axis is shown the percentage of historical households that have lateral (horizontal) extensions.

On the y-axis is shown the generational hierarchy index scores. This Figure is based on the maximum number of regions for which individual census data were available

(170 regions). Albania as an outlier was excluded from the analysis.
