# Supplementary material for: The shadow of the family: Historical roots of social trust in Europe
Source: PLoS One. 2024 Feb 12;19(2):e0295783. doi: 10.1371/journal.pone.0295783 (PMC10861049; doi:10.1371/journal.pone.0295783)
Supplement: Attachment — Submitted filename: Review_PLOS.docx [file pone.0295783.s009.docx]

Review: The Shadow of the Family - Historical Roots of Social Trust in Europe

This is a well-written paper that provides an interesting analysis of how historical family ties may be related to current levels of out-group trust. The setup of the paper is clear, and the structure, in overall, sound, although some sections would require reordering as it becomes at times unclear what the key argument is.

The key finding of the paper is convincing, namely that household size does not appear to matter as much as hierarchical organization within the family. The paper uses interesting datasets and addresses important questions. However, I see one key problem in their research design:

The life in transition survey is representative at the national level, but the authors use single observations also at lower levels of aggregation. I do not think this is a correct procedure, in terms of statistics. If the authors used any additional computation to overcome such sample design problems or if they are in possession of further details that allow treating the data as statistically representative at the subnational level, they must provide details on it. Otherwise, the reader remains under the impression that it is not viable to use the LITs data at the subnational level at all.

The multilevel analysis in its current form has some important weaknesses.

First, the authors need to report the ICC of their null model to provide the reader with the relevant information of how much variability in the dependent variable is actually explained by the higher level included, so level 2 and level3. Similarly, results should report the R2 at the different levels, to make the reader understand which percentage of the contextual effect is explained by the level-specific control variables you include. Second, the authors use history as a level in which the present is nested. This is not uninteresting, but deserves some discussion - in particular because the individuals in the present (and their characteristics) are meant to be nested within the characteristics derived from other individuals in the past. Such design needs to be properly understood which does not seem to be the case when the authors discuss causality at the end of the results section. They use the typical disclaimer that results do not suggest causality, but in a multilevel model higher levels dominate lower levels, so the causal directionality is implicit. Further, that paragraph seems to forget that past family ties are put into connection with present levels of trust, which also only allows for one directionality of causality.

The inclusion of the third level - with only 7 observations - is rather problematic and appears to be a shortcut for the inclusion of control-variables only. It would be better to eliminate level 3 and to include relevant controls at level 2 - disaggregating controls at the right level where possible.

Minor comments:

- In the introduction it remains unclear for some time in the text whether "the network" refers to in and/or out although that specification comes a bit later
- Similarly, the text refers to extendedness at the start and may want to clarify from the start whether this is just household size or the typical concept of extended family
- the anticipated conclusion on page 5 lines 62-65 is a bit unclear - the correlation between the two covariates has not yet been adressed at that point of the manuscript
- unclear for which reason the extended sample is introduced in the introduction if it does not serve the purpose of the manuscript (76-78) - could be made clear it is only used for robustness checks
- page 8 - 125-127 unclear
- neolocality in table 1 seems wrong - shouldn't it be more neolocality when the share of youngsters living only with their spouse is higher?
- On page 15 the description of the gender hierarchy index appears to mix indicators with opposite directionality (e.g. married women at young age and married women at older age) this is misleading and is actually in contrast with the specifics given in table 1 - authors could add at least a footnote assuring the reader that all directionalities are expected and to see table 1 for details - so phrase currently in lines 313-314 may be anticipated somehow
- Figure 4 and the entire treatment of "pure" horizontal and vertical linkage in which however horizontal ones appear to be comprised is unconvincing and seems to lead the reader astray. If vertical linkage comprise horizontal then this graph is not very helpful and the analysis dedicated to it somehow misleading.
- page 21: 432 - why is a weak correlation a sign for complementarity instead of substitutability? This should be derived from the sign, not from the strength of the correlation.
- the deepest explanation of isolative vs. cooperative comes only in the conclusion. Better treatment of the point should be anticipated in the manuscript.
