# Supplementary material for: The shadow of the family: Historical roots of social trust in Europe
Source: PLoS One. 2024 Feb 12;19(2):e0295783. doi: 10.1371/journal.pone.0295783 (PMC10861049; doi:10.1371/journal.pone.0295783)
Supplement: Attachment — Submitted filename: Response to reviewers RR3.docx [file pone.0295783.s011.docx]

Dear Editor,

We are very grateful for having been given the opportunity to revise and improve the paper further. We did our best to address remaining comments and made a professional proof-reading of the text by the native speaker. Below we present our responses to the comments. We start with the editorial comments and then present our responses to the reviewer’s comments. All comments are written in italics, followed by our responses written in normal font.

**Editorial comments**

*The authors primarily add footnote 10 to the manuscript, the statements in which might be difficult to comprehend for many readers. What do the authors mean by saying they are not "constructing indicators" at the subnational level and why does that make a difference? Can all readers be expected to know what primary sampling units are? - a term the authors use repeatedly without explaining it. The authors should at least make a clear statement whether unrepresentative data can have consequences for the internal or external validity of results, rather than a non-scientific statement like "this is not a crucial issue". The reviewer makes more suggestions how the issue could be addressed. The authors should evaluate and answer in their response which of these suggestions are useful to make their analysis more convincing or make its limitations transparent.*

We agree that those statements were ambiguous and we are grateful for the opportunity to clarify our argumentation. By the phrase “we don’t use LiTS data to construct any indicators at the subnational level” we wanted to emphasize that our study is not aimed to measure mean levels of trust at the subnational level. In other words, we are not interested in measuring the level of trust in PSU #1 or in PSU #24 or in any other particular PSU (Please, note that wherever possible LiTS uses local electoral units as PSU. In regions where local electoral territorial units were not available, Census Enumeration Districts or local authorities were used as PSUs. This clarification is added to the text.) If we would be interested in such measurements, then the non-representativeness of LiTS at subnational level, indeed, would be a crucial limitation.

Rather, in our paper we measure indicators trust of individuals living within PSUs and regress these indicators on historical family indicators at the PSU level. Our interest is to estimate the impact of historical family indicators on individual trust. So, the question is whether the non-representativeness of LiTS at the PSU level compromises our econometric findings, i.e., whether it causes any bias in the OLS estimates of the impact of historical family indicators on individual trust. We believe that it doesn’t cause any bias, and here’s why.

First of all, let us formulate why there may be a problem at all. The non-representativeness of LiTS at the PSU level means that the composition of the PSU samples differs from the composition of the corresponding PSU populations. This means that our dependent variable is, probably, measured with errors, as we would observe different values of trust within each PSU if PSU samples were representative. Therefore, the issue of non-representativeness is reduced to the issue of measurement errors in the dependent variable.

Next, as it is well known, measurement errors in the dependent variable bias the OLS estimator only if they are non-classical, i.e., if they are correlated with the explanatory variable (in this case the explanatory variable will be endogenous). If errors are not correlated with the explanatory variable, they are classified as classical and can be viewed just as a noise which doesn’t bias the OLS estimator [see the detailed review by Bound, Brown & Mathiowetz (2001) in Chapter 59 of the Handbook of Econometrics edited by J. Heckman and E. Leamer] Therefore, the key question is whether errors in our trust variable are classical or not.

To answer this question, we refer to the sampling procedure used in LiTS. It consists of two stages. First, for each country 50-75 PSUs are chosen from a larger country-specific list of PSUs with probability of selection proportional to the PSU size. Second, households which are surveyed are randomly chosen within each PSU. The random sampling of households within a PSU means that even if the sample doesn’t represent the proportions of the general population of PSU and trust values of the sampled individuals differ from the trust values of the population, these differences should be considered as random and thus not be correlated with any factors. In sum, possible measurement errors in our dependent variable due to the non-representativeness of LiTS at the subnational level should not bias our econometric estimates as those errors are random (by survey design).

Having said that, we acknowledge that possible concerns about the non-representativeness of LiTS’ at the regional level still may exist. Therefore, we made the effort to provide robustness checks to ease those concerns. First of all, we carefully considered the reviewer’s suggestion to look at the correlation between the dependent variable (trust) and historical family indicators at the country level (see our reply to the reviewer’s comments). Secondly, we provided an alternative robustness check, relying on external data source. We used “Values in the Russian Regions” survey (Almakaeva et al., 2019), which is representative at the regional level and uses exactly the same questions on trust as LiTS does. It appeared that the regional-level correlation between the out-group trust measure from LiTS and the out-group trust measure from that Russian survey is about 0.83***, which suggests that LiTS data provide rather good estimates for the mean levels of out-group trust at the subnational level, at least for one country, Russia. We add to the revised version of the paper a paragraph where we provide all our arguments and mention this robustness check (lines 456-470).

Concerning internal validity, the argumentation presented above implies that our findings are valid *internally* (as our research design and analysis answer the research question without bias).

*Beyond the reviewer's comments, I would ask the authors to fix equation one. Also (vectors of) control variables are accompanied by (vectors of) parameters to be estimated. I don't understand why the authors speak in this particular spot of "historical countries(sic!) fixed effects". These are simply country fixed effects, right?*

Yes, these are country fixed effects. However, historical and contemporary countries differ to some extent. For example, in the historical census lists Scotland is mentioned separately from England and Wales. The Hungarian part of the Habsburger empire included the present-day territories of Croatia and Slovakia. Therefore, historical countries FE differ from (contemporary) countries FE. We include FE for historical countries but not FE for the contemporary countries to better control for omitted variables which may affect both historical family structures and out-group trust. Any contemporary societal characteristics, which may be omitted for the model, don’t affect historical family structures but rather may be influenced by them.

*The manuscript still includes many mistakes. "e.g." includes two punctuation marks and is regularly followed by a comma.*

This problem was fixed.

*One weblink in footnote 8 includes a space.*

The space was removed.

*Lateral is not capitalized.*

We use capital letters when Lateral means the variable name, while lower-case is used when lateral is an adjective (lateral extensions). We double-checked this.

*"in Appendix" requires an article.*

We fixed that.

*I see at least three different citation styles used in-text.*

Now we stick to one citation style.

*What do the authors mean by "Albania, Croatia, France, Hungary, Romania, Slovakia, Sweden, and United Kingdom (which correspond to five historical states: Albania, France, Great Britain and Wales, Hungary, Romania, Scotland, and Sweden)."? And how do they use the term Great Britain (especially w.r.t. Scotland and Wales, which appear to be separate)?*

As we mentioned earlier, historical and contemporary states do not entirely overlap. Contemporary Croatia, Hungary and Slovakia were part of the Habsburger empire (Hungarian part). In the 19^th^ century England and Wales had a separate census from Scotland. Great Britain was just a misprint, we meant England. We corrected it in the revised version and used “the Hungarian part of the Habsburger empire” instead of historical Hungary (see lines 83-84).

**Reviewer’s comments**

Old comments

**Reviewer’s comment 1.1 (original)**

*The Life in Transition Survey is representative at the national level, but the authors use single observations also at lower levels of aggregation. I do not think this is a correct procedure, in terms of statistics. If the authors used any additional computation to overcome such sample design problems or if they are in possession of further details that allow treating the data as statistically representative at the subnational level, they must provide details on it. Otherwise, the reader remains under the impression that it is not viable to use the LITs data at the subnational level at all.*

**Our response 1.1 (previous)**

We agree that this is a methodological limitation of our study, which we now mention in the revised version (see footnote 10 at p.19). However, we believe that the non- representativeness of LiTS at the subnational level it is not a crucial issue in our case as we don’t use LiTS data to construct any indicators at the subnational level. Rather, we assign historical indicators taken from external data sources to all individuals living within the same primary sampling units (PSU). In this case, a two-stage quasi-random sampling procedure used in LiTS (first, for each country 50-75 PSUs are chosen from a larger country-specific list of PSUs with probability of selection proportional to the PSU size, and, second, households are randomly chosen within each PSU) should prevent any systematic bias in the composition of PSU samples and thus should guarantee that our estimated coefficients are free of the sample-selection bias. The representativeness of LiTS at subnational level would be a pleasant bonus in this case, but it shouldn’t affect the econometric estimates.

Additionally, we would like to note that the literature contains plenty published examples which follow a similar procedure as we do: first, match subnational level indicators to microdata from cross-national surveys which are not representative at the subnational level, and, second, estimate the impact of those indicators on some individual level dependent variables. For instance, Ajzenman et al., (2022) in their recent study also use LiTS data to show that the exposure to mass transit migration measured at the PSU level (the distance from the locality to the closest transit migrant route) affects individual entrepreneurial activity and anti-migration sentiments. Schulz et al., (2019) assigns historical indicators calculated at the level of contemporary subnational regions to the European Social Survey (ESS) data which are not representative at the subnational level as well. Duranton et al. (2009) derive their measures of social capital and income inequality at the NUTS-2 level from the European Community Household Panel (ECHP) microdata, which are not representative at the subnational level. This last example, probably, is vulnerable to the problem of the non-representativeness at the subnational level, but this is not the case of our paper.

**Reviewer’s comment 1.2 (new)**

*I do not think the argumentation is satisfying. Just because other authors “got away” with a wrong research design does not justify not taking care of it. I do think the following is still necessary:*

*-       some more findings, which could take the form of a robustness test in which you e.g. correlate historical subnational data with national present trust-values, or some more detailed descriptive statistics such as a map of the dependent variable at the subnational level that can be compared to your already produced map with historical data. You can opt for a graph that visually pictures the correlations given the number of observations of countries is low*

*-       such findings should be commented in order to assess whether the lack of representativty at the subnational level of the lis design risks altering any of your findings. It is important to do so as the dependent variable relies on that sample.*

*-       if any of your findings change, please make sure you report the issue also in the section dedicated to the limitations of the study*

*Please note that your figure 6 right now has a note that stresses within-country variability as key factor for your findings. This stresses the need for greater precision in dealing with this important limit of the data. I recommend revising that note, by the way because it currently does not make too much sense but reports a contradictory sense.*

**Our response 1.2 (new)**

We accept that our argumentation was unclear and non-convincing. Let us explain better and in more details why we believe that the non-representativeness of LiTS at the subnational (PSU) level should not compromise our econometric findings.

First of all, let us formulate why there may be a problem at all. The non-representativeness of LiTS at the PSU level means that the composition of the PSU samples differs from the composition of the corresponding PSU populations. This means that our dependent variable is, probably, measured with errors, as we would observe different values of trust within each PSU if PSU samples were representative. Therefore, the issue of non-representativeness is reduced to the issue of measurement errors in the dependent variable.

Next, as it is well known, measurement errors in the dependent variable bias the OLS estimator only if they are non-classical, i.e., if they are correlated with the explanatory variable (in this case the explanatory variable will be endogenous). If errors are not correlated with the explanatory variable, they are classified as classical and can be viewed just as a noise which doesn’t bias the OLS estimator [see the detailed review by Bound, Brown & Mathiowetz (2001) in Chapter 59 of the Handbook of Econometrics edited by J. Heckman and E. Leamer] Therefore, the key question is whether errors in our trust variable are classical or not.

To answer this question, we refer to the sampling procedure used in LiTS. It consists of two stages. First, for each country 50-75 PSUs are chosen from a larger country-specific list of PSUs with probability of selection proportional to the PSU size. Second, households which are surveyed are randomly chosen within each PSU. The random sampling of households within a PSU means that even if the sample doesn’t represent the proportions of the general population of PSU and trust values of the sampled individuals differ from the trust values of the population, these differences should be considered as random and thus not be correlated with any factors. In sum, possible measurement errors in our dependent variable due to the non-representativeness of LiTS at the subnational level should not bias our econometric estimates as those errors are random (by survey design).

We very much hope that our argumentation is more clear now. We believe that this also explains the widespread use of a research design similar to ours in many quantitative studies.

Having said that, we acknowledge that possible concerns about the non-representativeness of LiTS’ at the regional level still may exist. Therefore, we made the effort to provide robustness checks to ease those concerns.

First of all, we carefully considered the reviewer’s suggestion to look at the correlation between the dependent variable (trust) and historical family indicators at the country level. If we understand well, the idea behind that suggestion is that that correlation could indicate what the correlation at the subnational level could be if LiTS would be representative at the subnational level (since LiTS is representative at the national level). While we like this idea, unfortunately, it doesn’t work in practice. First, we have only eight country-level observations, which makes any estimates very imprecise. Second, and more important, countries are too wide and heterogeneous units. Averaging trust and historical family indicators erases almost all meaningful variation in these variables. The variation that we use for identification in our paper is subnational and comes from *within* countries (as we include country FE). Therefore, we are afraid that the correlation obtained at the country level hardly may be indicative of the correlation at the subnational level.

While trying to invent alternative checks, we turned to the survey “Values in the Russian Regions” which was conducted in January–February 2019 (Almakaeva et al., 2019). This survey is representative at the regional level and uses exactly the same questions on trust as LiTS does (these data will be available as a supplementary material to our paper). It appears that the regional-level correlation between the out-group trust measure from LiTS and the out-group trust measure from that Russian survey is about 0.83***, which suggests that LiTS data provide rather good estimates for the mean levels of out-group trust at the subnational level, at least for one country, Russia. We mention this check in the revised version of the paper.

We add to the revised version of the paper a paragraph where we provide all our arguments (lines 456-470).

Concerning Fig.6, we revised that note. However, we cannot eliminate it completely as it explains to the readers how Fig. 6 is constructed. It reflects the within-country variation, as exactly that variation is used in our regressions for identification (as historical country fixed effects which remove all between-country variation are included in our regressions).

**Comment 2.1 (original)**

*the deepest explanation of isolative vs. cooperative comes only in the conclusion. Better treatment of the point should be anticipated in the manuscript.*

**Response 2.1 (new)**

In the revised version, we introduce the explanation of the “isolative” and “cooperative” components in the introduction and then we elaborate it in the hypotheses section.

**Comment 2.2 (new)**

*I saw the new text you included but still think it remains vague. Please make an effort to improve it. Line 72-73 “isolative” is still unclear as it seems to describe also horizontal and not vertical – please clarify this a little bit more. My suggestion is to rework the paragraph starting at line 195. Can you summarize this logic in a diagram? and refer back to it in your conclusions? The point seems to be relevant for your findings but the exposition in the paper currently does not allow to properly grasp the implications.*

**Response 2.2. (new)**

You are completely right: both horizontal and vertical family extensions mean a larger number of relatives and lead to more self-sufficiency of the household and less need for social interactions beyond the kin group. We tried to make it more clear in the revised version (see line 62). Additionally, we reworked that paragraph and added a special Fig.1 “Theoretical mechanisms of the effect of family extendedness on out-group trust.”, where we illustrate the negative and the positive pathways running from family extendedness to out-group trust**.** We refer to the “isolative” and “cooperative” components of family extendedness in the concluding section.

Fresh comments

*line 129 “gender equality stimulated…” are these just co-evolving factors or has causality been proved here? If yes, please cite relevant literature.*

We agree that the causal impact of gender inequality on other variables hasn’t been proved (as it is extremely difficult to find a valid instrument), this we corrected that sentence.

*167-171: argumentation is VERY speculative.*

We revised this paragraph (see lines 171-183). We believe that persistence of family structure is not the only institutional channel supporting persistence of trust. There might be persistence or path-dependence of other societal institutions that may have an effect on trust like good government, effective law, or participatory institutions. All these institutions provoke social trust by stimulating cooperation between people and making it less risky. Consequently, trust persistence may rely on the continuity of these institutions.

*Is Putnam’s argument regarding horizontality among peers really isomorphically valid also at the family-level?*

We believe that Putnam’s argument is applicable to the family level as well. It is even more important to elaborate impartial norms of interaction between relatives of equal social status within a household because otherwise they would not be able sustain regular daily communications which they cannot avoid while living under one roof. We added this argumentation to the text, see lines 215-218 in the revised version.

*Lines 202-205 simply extend the argument of trusting each other in similar conditions (blood-link) to ones in which the blood-link is missing.*

We provided a link to the paper by Welzel and Delhey (2015) where the authors state that out-group trust grows to a large extent out from in-group trust because people should learn to trust someone before they can trust everyone. Their theory justifies the extension of our argument of trusting each other within the family to trusting strangers. See lines 221-223 in the revised text.

*How about mentioning the role that the share of servants in the family plays for your key argument?*

Thank you for this suggestion. The presence of servants in the household is responsible for communications with the out-groups on a daily basis and for the formation of mutually acceptable social norms. We added this paragraph to the text, see lines 224-228.

*Can you please add the correlations of factors with the latent factors you extrapolate from the PCA? that is relevant to see as a reader.*

We added Table A4 (in the Appendix) with the results of two separate Principal Components Analyses that we performed to create generational and gender hierarchy indices. Numbers indicate the strength of correlation of each variable with the eigenvector of the first principal component for each PCA.

*When you mention that a higher share of female-headed hh stands for greater independence, please consider that this can be a phenomenon due to war or specific migratory patterns.*

We agree with this comment and made a robustness check. We re-constructed the gender hierarchy index without this item and run the regressions with such an index. Table A5 in Appendix shows that the results do not change.

*I found it rather odd to discuss hunter-gatherers in the context of European data in the last centuries. While I think it is ok to include the variable stressing some kind of geographical determinants on culture, I think the current discussion around the variable in the text is misplaced.*

Thank you for this remark. We tried to clarify this issue in the revised text (see lines 440-446). Welzel et al., (2021) stress the fact that territories well suited for hunting and gathering adopted agriculture several thousand years later than the first adopters in the Middle East. According to their theory, it created favorable conditions for the future Industrial Revolution and left an imprint on contemporary social values and institutions. In light of this evidence we consider it is appropriate to use land suitability for hunting and gathering as a control variable.

*Figures 4 and 5: you need to exclude the outlier Albania at least to give a better perspective on the results you find. In this moment the two figures are not intelligible because of that outlier. You can include either of the two versions in the appendix.*

We provided these figures without Albania in Appendix (see Fig. A4 and Fig. A5).

*I think you need to be much more precise in data labelling throughout. Which variables are historical which ones are current? E.g. in figures 4 and 5 it is not clear.*

We changed the variable labels in Figures 4 and 5 so that it is now clear that we are using historical data.

*Lines 8-10: you start with “evidently…” but there could by many other reasons for such finding.*

Unfortunately, we didn’t find “evidently…” in those lines.

*524-525: Should’nt it say “decreases”?*

Sure, it means decreases. We corrected this.

*The limitations section is ok but rather hidden in the text. I suggest to expand it and to provide a title for it.*

Following your suggestion, we expanded this section and provided a title for it.

*While I recognize some effort in the revision, I found the paper harder to read, this time. Somehow I have the impression the authors just want to push their claims with too much vehemence. But the statistical analysis, although implemented with rigour is not able to lift all doubts regarding those claims. This has to do with data typology, number of observations and the complexity of the subject and time frame investigated.*

*I am in favour of showing this kind of work, but I would welcome a bit more humility in the exposition and the claims made, especially when the subject is easily transposed to larger levels e.g. formal and informal institutions. I also strongly advise to pay more attention to the limitations that an analysis like this is clearly confronted with, in order to tease out the most reliable evidence.*

Following your comments we tried to make the limitations of our study more transparent to readers and smoothed some claims made. We spent a lot of effort and did our best to provide a validation test for the subnational LiTS data (see the response to your previous comments). Unfortunately, this limitation still remains and we mention it in our limitation section.
